# The RING-H2 protein RNF11 is overexpressed in breast cancer and is a target of Smurf2 E3 ligase

**DOI:** 10.1038/sj.bjc.6601301

**Published:** 2003-10-14

**Authors:** V Subramaniam, H Li, M Wong, R Kitching, L Attisano, J Wrana, J Zubovits, A M Burger, A Seth

**Affiliations:** 1 Laboratory of Molecular Pathology and Molecular and Cellular Biology Research, Sunnybrook and Women's College Health Sciences Centre, CIHR Group in Matrix Dynamics, University of Toronto, Toronto, Ontario, Canada; 2 Department of Biochemistry, Department of Laboratory Medicine and Pathobiology, University of Toronto, Toronto, Ontario, Canada; 3 Samuel Lunenfeld Research Institute, Mount Sinai Hospital, University of Toronto, Toronto, Ontario, Canada M5S 1A8

**Keywords:** RNF11, ubiquitination, Smurf2, TGF*β* signalling

## Abstract

The breast cancer-associated T2A10 clone was originally isolated from a cDNA library enriched for tumour messenger ribonucleic acids. Our survey of 125 microarrayed primary tumour tissues using affinity purified polyclonal antibodies has revealed that corresponding protein is overexpressed in invasive breast cancer and is weakly expressed in kidney and prostate tumours. Now known as RNF11, the gene encodes a RING-H2 domain and a PY motif, both of which mediate protein–protein interactions. In particular, the PPPPY sequence of RNF11 PY motif is identical to that of Smad7, which has been shown to bind to WW domains of Smurf2, an E3 ubiquitin ligase that mediates the ubiquitination and degradation of the TGF*β* receptor complex. Using various mutants of RNF11 in GST pulldown and immunoprecipitation assays, we found that RNF11 interacts with Smurf2 through the PY motif, leading to ubiquitination of both proteins. Smurf2 plays an active role in the repression of TGF*β* signalling, and our data indicate that overexpression of RNF11, through its interaction with Smurf2, can restore TGF*β* responsiveness in transfected cells.

Breast malignancy at the molecular level is partly due to genetic mutations and deletions that result in the alteration of expression and function of various cellular genes. Although progress has been made in characterising some of these genes, the specific molecular mechanisms underlying many of the cellular pathways remain to be elucidated. Once identified, such differentially expressed genes will be useful in monitoring disease progression and understanding the molecular mechanisms of tumour development. In addition, the molecular definition of new genes involved in breast tumours will yield novel targets for new therapeutic strategies.

The T2A10 gene was originally cloned as a partial cDNA from a library enriched for breast tumour messenger ribonucleic acids (mRNAs) (Burger *et al*, 1998). Subsequently, we extended this cDNA sequence ‘*in silico*’ using overlapping expressed sequence tags (ESTs) and cloned a full-length copy DNA (cDNA) corresponding to a 2.4 kilobase (kb) mRNA (Kitching *et al*, 2003). We found its open reading frame (ORF) to be identical to the human ring-finger 11 (RNF11) protein (Seki *et al*, 1999). The predicted amino-acid sequence for the longest RNF11 ORF encodes a RING-H2 domain and a PY motif, both of which mediate protein–protein interactions for proteins involved in signal transduction pathways and ubiquitin (Ub)-mediated proteolysis. PY motif sequences are found in a variety of regulatory proteins including the AP-2 transcription factor, p53BP-2, RasGAP, and Smads 2, 3 and 7 (Yagi *et al*, 1999). Each of these proteins binds to specific WW domain containing proteins, such as YAP, WWOX, Smurf1, Smurf2 and NEDD4 (Yagi *et al*, 1999; Kavsak *et al*, 2000; Winberg *et al*, 2000; Conaway *et al*, 2002; Suzuki *et al*, 2002; Yendamuri *et al*, 2003). Ubiquitination- and proteasome-mediated degradation of proteins is an important step in cellular signalling, and disruption of this process has been implicated in many human diseases including cancer (Hershko and Ciechanover, 1998). Similar to RNF11, Smad proteins (Smads 2, 3 and 7) contain PY motifs that act as substrates and adaptors for the Smurf2 E3 Ub ligase in the transforming growth factor *β* (TGF*β*) signalling pathway (Mehra and Wrana, 2002). TGF*β* is a multifunctional peptide that controls proliferation, differentiation and other functions in normal and transformed cells. TGF*β* signalling molecules have been shown to have a crucial role in breast and other cancers (Attisano and Wrana, 2002).

Here, we investigated RNF11 protein expression in an array of 125 human primary tumours, including mammary carcinomas, and studied potential protein binding partners. The PY motif of RNF11 suggests a link to TGF*β* signalling through interactions with the WW-binding domains of Smurf2. The functionality of this motif was tested by *in vivo* binding and ubiquitination assays with Smurf2. Finally, we demonstrate that RNF11 overexpression can preserve TGF*β* receptor responsiveness in mammalian cells.

## METHODS

### Antibodies

C14N polyclonal antibody was raised in rabbits against a synthetic hexadecapeptide sequence derived from the RNF11 C-terminal region conjugated to keyhole limpet haemocyanin (KLH). Rabbit IgG polyclonal antibodies were purified on a peptide affinity column. FLAG (M2), *β*-actin and glutathione-*S*-transferase (GST) antibodies were purchased from Sigma. Haemagglutinin (HA) antibody (12CA5), which recognises a nonapeptide sequence (YPYDVPDYA), derived from the influenza haemagglutinin protein, was from Roche. Anti-polyhistidine (HIS-tag) antibody was from R&D systems. Protein A-sepharose beads were from Sigma and gluthathione sepharose beads were from Amersham Pharmacia.

### Tumour tissue microarray (TMA) and immunohistochemistry (IHC)

Tumour cases were selected from the archives of the Department of Anatomic Pathology, Sunnybrook and Women's College Health Sciences Centre. Following selection of tumour blocks and verification of the diagnosis, tissue was anonymised by stripping all identifiers from each case. Areas of cancer and normal tissue elements were marked on haematoxylin and eosin (H&E) sections by a pathologist (JZ). A multi TMA was assembled consisting of 20 breast, 15 prostate, 17 bladder, 16 colon, 11 head and neck, seven lung, 17 renal and 22 pancreas carcinomas by punching 0.6 mm tissue cores from the donor block and transferring them to a recipient block. A total of 125 cases were arrayed in duplicates using two blocks for each case. The array was essentially constructed and transferred onto slides following the TMA methodology as described (Kononen *et al*, 1998).

Tumour-specific RNF11 expression was monitored by IHC using the Histostain SP rabbit kit with DAB substrate from Zymed (San Francisco, CA, USA). The primary RNF11 antibody (0.4 mg ml^−1^, 1 : 125 dilution) was used to probe TMA sections processed and evaluated as reported previously (Landberg *et al*, 2001). For negative controls, RNF11 antibody was preincubated with the RNF11 peptide (10 : 1 ratio with antibody) used for immunisation 1 h prior to the reaction with the TMA. Results are representative of three independent experiments.

### Vectors

Human Flag-Smurf2 and HA-tagged deletion mutants of Smurf2, HA-tagged Smad7, p3TP-Lux, polyhistidine (HIS) tagged Ubc3, UbcH5a, UbcH5b, UbcH5c, and HA-tagged Ub are as described by Kavsak *et al* (2000) and Bonni *et al* (2001). RNF11 and the engineered mutants of RNF11 were derived from the RT–PCR cloned ORF of RNF11 in vector plasmid pCMV-T2C using the Quikchange Site Directed Mutagenesis Kit (Stratagene). For the RNF11-*mt*PY construct, the tyrosine at position 40 was substituted to alanine, and in the RNF11-*mt*RING construct the cysteines at positions 89 and 93 were mutated to serine 89 and serine 93. The RNF11-*mt*Double mutant has both the PY and the Ring mutation.

### Cell lines and transfection assays

HEK293T is a simian virus 40 (SV40) large T-antigen transformed human embryonal kidney cell line that can be transiently transfected, and is therefore commonly used to study protein–protein interactions by immunoprecipitation (Di Guglielmo *et al*, 2003). The HEK293T cell line was maintained in high glucose DME medium (Gibco) containing 10% FBS without antibiotics in 5% CO_2_ atmosphere. HepG2 cells were maintained in minimal essential medium (MEM) (Gibco) containing Earle's salts, L-glutamine, 1 mM sodium pyruvate, 0.1 mM nonessential amino acids and 10% FBS in 5% CO_2_ atmosphere. For transfections, 1 × 10^6^ cells were plated in triplicate in 100-mm dishes and grown overnight, medium was changed 2 h prior to transfection. Each plasmid DNA (2–5 *μ*g) was mixed with 250 mM CaCl_2_ and 2 × HBS (280 mM NaCl, 50 mM HEPES, 1.5 mM sodium phosphate (pH 7.05)) and incubated at room temperature for 15 min. The precipitate was then added to exponentially growing cells (Hoodless *et al*, 1996). One of at least three similar experiments is shown.

### Immunoprecipitation and Western blots

Cell lysates were immunoprecipitated (IP) with mouse anti-FLAG, anti-GST or anti-HA antibody, and Western blots were performed as described (Winberg *et al*, 2000). Briefly, 42–48 h post-transfection, cells were lysed in 900 *μ*l of lysis buffer (50 mM Tris pH 7.6, 150 mM NaCl, 0.1% NP-40) containing protease inhibitors. Cell lysates were incubated with antibodies for 1 h at 4°C. Protein A-sepharose beads were added to the lysates, which were then incubated for 30 min at 4°C, and the resulting immunoprecipitates were washed with lysis buffer three times. Immunoprecipitates and aliquots of total cell lysates were separated by sodium dodecylsulphate–polyacrylamide gel electrophoresis (SDS–PAGE) and transferred to a Hybond-P membrane (Amersham). Antibody reactive proteins were detected using horse radish peroxidase (HRP)-conjugated secondary antibodies and visualised by chemiluminesence (Amersham). Representative Western blots from one of at least three similar experiments are shown. C14N antibody competition with the immunising peptide was carried out on duplicate Western blots using a 10 : 1 ratio of peptide to antibody.

### Transcriptional response assay

HepG2 is one of the tumour lines that retain TGF*β* responsiveness in culture and is therefore used here (Di Guglielmo *et al*, 2003). For TGF*β* inducible luciferase reporter assays, HepG2 cells were transiently transfected with the reporter plasmid (3TP-Lux), pCMV-*β*-gal and the indicated constructs or with the empty vector alone. To induce the luciferase reporter, cells were treated overnight with 100 pM of TGF*β* as described (Hayashi *et al*, 1997). Luciferase activity in cell lysates was measured using the luciferase assay system in a Berthold Lumat LB 9501 luminometer (Promega).

## RESULTS

### RNF11 protein is overexpressed in breast tumours

RNF11 protein expression in primary tumours was monitored by IHC using affinity purified anti-human RNF11 polyclonal antibody on a TMA composed of duplicate punches from 125 primary tumours representing eight different histologies. IHC results for the TMA are summarised in Table 1

**Table 1:** Immunohistochemical staining with RNF11 antibody in 125 microarrayed tumour tissue sections; duplicate micropunches of each tumour were remounted and sectioned to produce the microarray

**Tumour type**	**No. evaluated**	**Positive (%)**	**IHC staining intensity**
Breast	20	90	+++
Bladder	17	31	++
Colon	16	47	++
Head and neck	11	63	+
Kidney	17	11	+
Lung	7	57	+
Pancreas	22	78	++
Prostate	15	10	+

**Figure 1:**
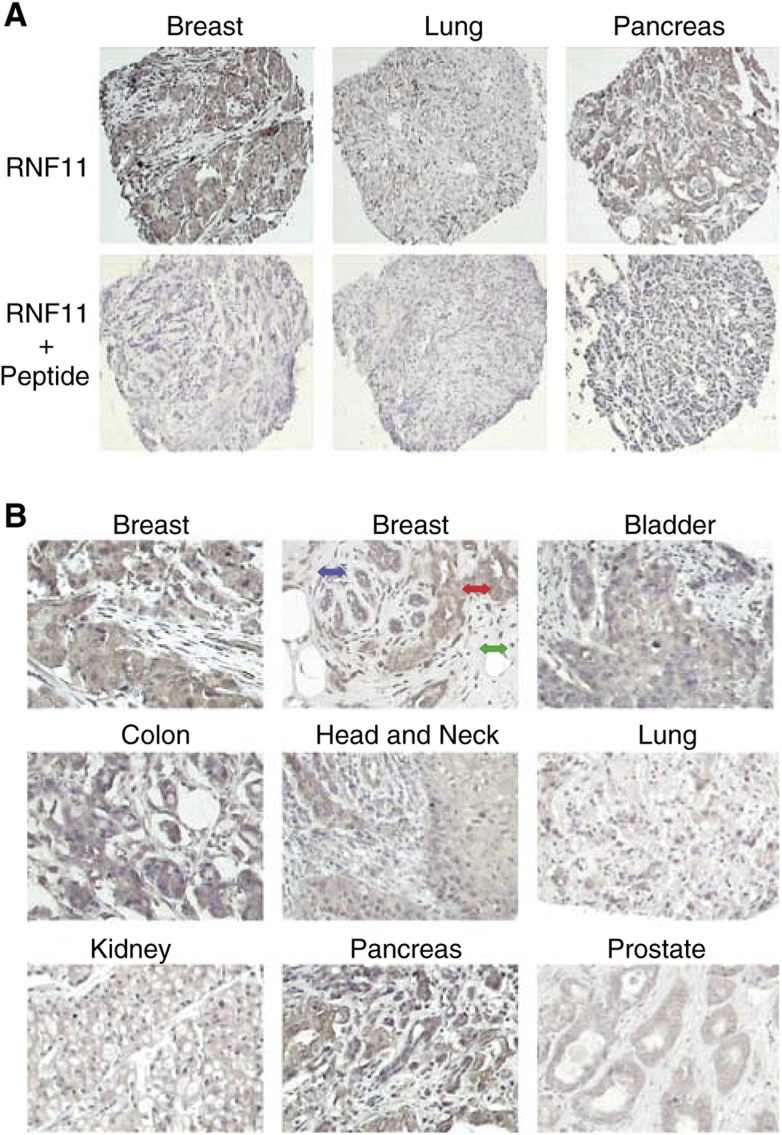
RNF11 protein expression in tumour tissues. (**A**) TMA sections stained with anti-RNF11 antibody and anti-RNF11 antibody preincubated with synthetic peptide used for immunisation (magnification × 40). (**B**) Representative examples of RNF11-positive tumour tissues from eight different histologies as indicated on top of the each picture. Invasive breast carcinoma cells (red arrow) are strongly RNF11 positive as compared to surrounding stroma (green arrow) and normal breast glands (blue arrow) (magnification × 200).

+=weak; ++=intermediate; +++=strong to very strong immunoperoxidase/DAB intensity (brown).

, and indicate that RNF11 is predominantly expressed in the cytoplasm of cancer cells (Figure 1A, B

, Table 1). Staining of primary breast tissues with anti-RNF11 antibody showed that the corresponding protein is high in cancer cells, but low in normal ductal structures and absent in surrounding stroma (Figure 1B). Strong cytoplasmic RNF11 staining was found in >90% of the invasive breast cancer cases, in adenocarcinomas of the pancreas (78%), colon cancer (47%) and in bladder tumours (31%) (Table 1, Figure 1). Table 2

**Table 2:** RNF11 Expression in relation to clinicopathological parameters in breast cancers

**Histology**	**#**	**RNF11**	**BR grade**	**ER**	**PR**	**LN**
INV, ductal NOS	18	2.26±0.35	II–III	14+/4−	7+/11−	7+/8−/3NK
INV, ductal papillary 2	2	2±1	II–III	1+/1−	2−	2−

INV=invasive; #=number of cases; BR grade=Bloom–Richardson grading system; ER=oestrogen receptor status; PR=progesterone receptor status; LN=lymph node status; NK=not known; RNF11=mean staining intensity±s.d. of IHC score.

summarises the RNF11 staining with clinicopathologic features such as oestrogen and progesterone receptor status (ER and PR), Bloom–Richardson grading (BR grade), lymph node (LN) status and histology.

Modest RNF11 expression was seen in 63% of the head and neck tumours, and 57% of the lung tumours, whereas renal and prostate carcinomas stained only weakly positive for RNF11 protein (∼10%) (Figure 1B, Table 1). The specificity of RNF11 staining in the arrayed tumour tissues was confirmed by probing control sections with RNF11 antibody that had been preincubated with the hexa-decapeptide used for immunisation (Figure 1A). Although RNF11 protein expression was predominantly located in the cytoplasm in all eight tissue types on the TMA (Figure 1), some nuclear RNF11 accumulation was observed in lung, renal, and head and neck cancers (Figure 1B).

### RNF11 interacts with Smurf2 through the PY motif

**Figure 2:**
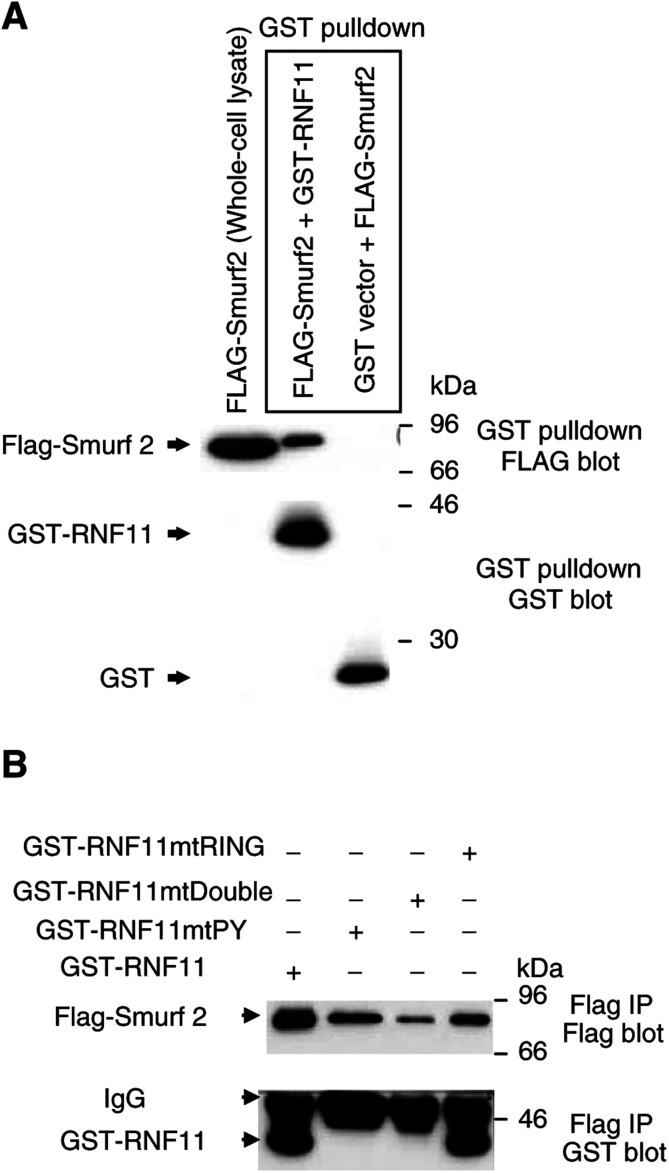
RNF11 interacts with Smurf2 through the PY motif. (**A**) FLAG and GST Western blots of whole-cell lysates and GST pulldowns or (**B**) FLAG immunoprecipitates from HEK293T cells transiently transfected with the Flag-Smurf2 expression vector and wild-type GST-RNF11 or its mutants using the calcium phosphate precipitation method.

In order to test RNF11 interaction with Smurf2, we transiently transfected the HEK293T cell-line with GST-tagged RNF11 and three mutant RNF11 constructs together with a FLAG-tagged Smurf2 expression vector (Figure 2

). The GST-tagged RNF11 mutants included RNF11-*mt*PY in which the tyrosine of the PY motif was mutated to alanine, RNF11-*mt*Ring, where the first two cysteines in the RING-finger domain were replaced by two serines and the RNF11-*mt*Double having both mutations. GST pulldown of lysates from cultures cotransfected with Flag-Smurf2 and wild-type GST-RNF11 isolated a protein corresponding to Flag-tagged Smurf2 (as shown by anti-Flag Western blot), but not from lysates of cells cotransfected with Flag-Smurf2 and the GST-vector alone (Figure 2A). This result was confirmed by anti-Flag immunoprecipitation and GST immunoblot of lysates from HEK293T cells transfected with Flag-Smurf2 and each of the GST-RNF11 constructs, the *wt* RNF11, the PY mutant, the RING-H2 mutant or the double mutant (Figure 2B). GST-RNF11 bands were observed only with wild-type RNF11 and the Ring mutant but not with the RNF11-*mt*PY or RNF11 double mutant, indicating that RNF11 interacts with Smurf2 through its PY motif.

### Smurf2 interaction with RNF11 requires WW domains 2 and 3

**Figure 3:**
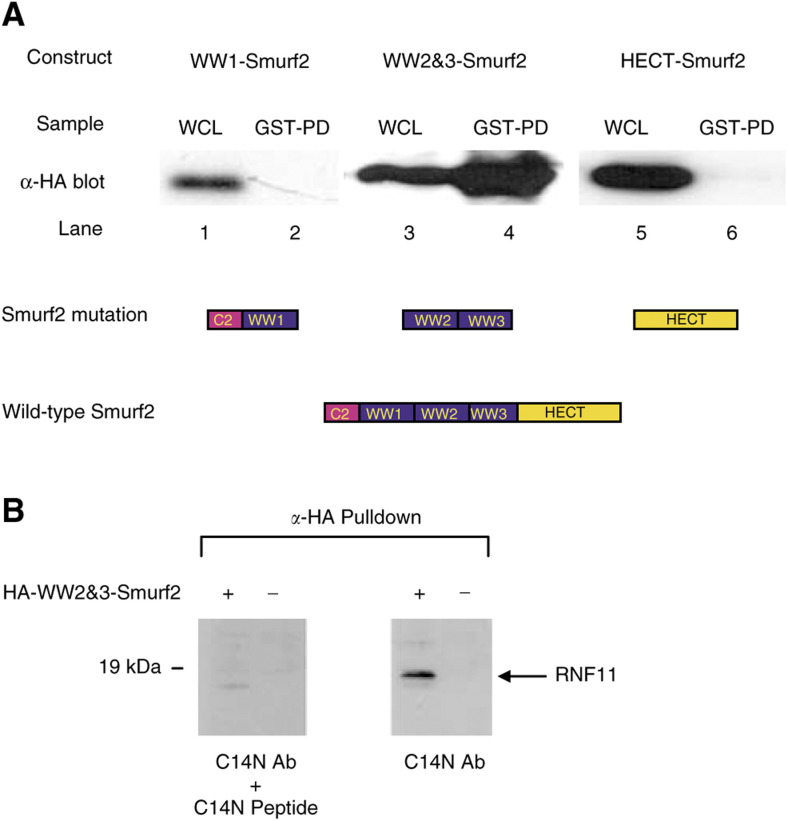
GST–RNF11 interactions with Smurf2 mutants. WW1-Smurf- is a mutant of Smurf2 lacking WW-binding domains 2 and 3. WW2&3 is a deletion mutant of Smurf2 lacking the C2, WW1 and HECT domains. HECT-Smurf2 is a deletion mutant of Smurf2 containing only the HECT domain: (**A**) top panel: various domains in Smurf2; middle panel: HA immunoblots of whole-cell lysates (WCL) of cells transfected with HA-tagged Smurf2 mutants and GST-RNF11 and GST pulldown (GST-PD) of the transfected cell lysates; bottom panel: schematics of the Smurf2 mutants. (**B**) Endogenous RNF11 interacts with WW domain of Smurf2. Chemiluminescent detection of endogenous RNF11 with anti-RNF11 antibody in the presence (left panel) and absence (right panel) of the immunizing peptide on Western blots containing proteins pulled down by anti-HA agarose beads from cell extracts prepared from 293T cells transfected by HA-Smrf2 WW2&3 construct.

In order to identify which of three WW domains in Smurf2 could mediate RNF11 binding, HEK293T cells were cotransfected with expression vectors for GST-RNF11 and each of three HA-tagged Smurf2 deletion mutants (Figure 3A

). The HECT-Smurf2 mutant contains no WW domains, and GST pulldown followed by anti-HA Western blot did not resolve a band corresponding to HECT-Smurf2, indicating that in the absence of WW domains, Smurf2 does not interact with RNF11 (Figure 3A, lane 6). No HA-tagged Smurf2 band is seen in HA blots of GST pulldowns from cells cotransfected with GST-RNF11 and WW1-Smurf2, indicating that WW domain 1 is not sufficient for Smurf2–RNF11 interaction (Figure 3A, lane 2). However, when Smurf2 deletion mutant containing WW2 and WW3 domains was tested, a band corresponding to WW2&3-Smurf2 protein can be seen on the HA Western blot after GST pulldown using GST-RNF11 protein (Figure 3A, lane 4). In all three cotransfections, the anti-HA Western blot of whole-cell lysates shows that mutant HA-tagged Smurf2 proteins were available for pulldown mediated by interactions of GST-RNF11 and HA-Smurf2 proteins (Figure 3A, lanes 1, 3 and 5).

Endogenous RNF11 interaction with Smurf2 WW domains was examined by anti-RNF11 Western blot after HA pulldown of the WW2&3-Smurf2 construct from transfected cells (Figure 3B). The C14N anti-RNF11 antibody recognises an 18 kDa band that is not visible in the presence of the immunising peptide.

### RNF11 interacts with UbcH5s but not with Ubc3

**Figure 4:**
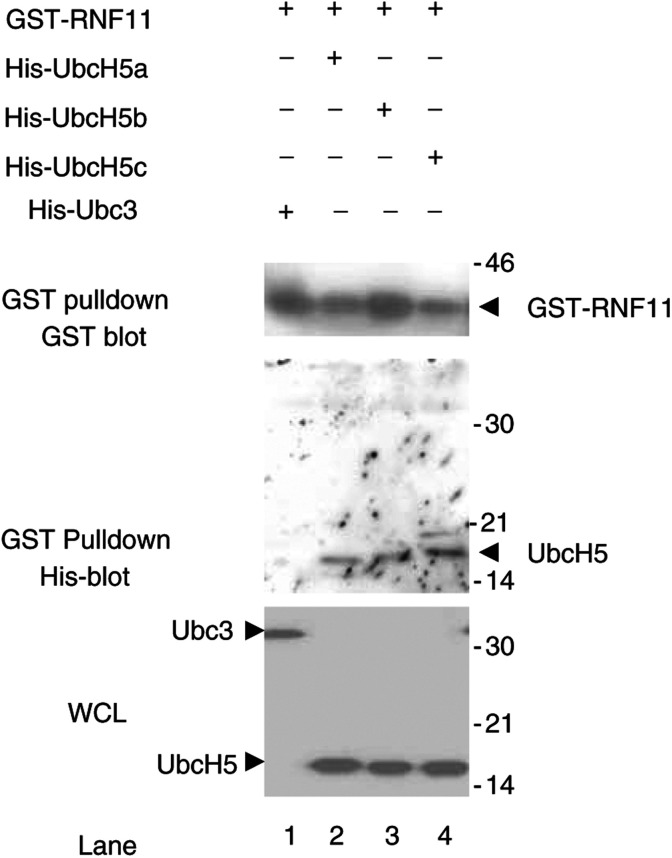
RNF11 interacts with UbcH5s but not with Ubc3. GST and His-Tag Western blots of whole-cell lysates and GST pulldowns from 293-T cells cotransfected with GST-RNF11 and each of the His-tagged E2 protein expression vectors as indicated.

Most ubiquitination complexes require an E2 conjugating protein to provide the Ub moiety for the E3 ligases or multisubunit E3 ligase complexes (Freemont, 2000; Joazeiro and AM Weissman, 2000; Thrower *et al*, 2000; Zheng *et al*, 2002). We found that Smurf2 E3 ligase interacts with RNF11 through its WW domains (Figures 2 and 3). RNF11 also contains a RING-H2 domain, some examples of which are known to bind ubiquitin conjugating (Ubc) proteins involved in ubiquitination (Freemont, 2000; Thrower *et al*, 2000; Zheng *et al*, 2002). This suggested to us that one or more Ubc proteins might interact with RNF11, and this was tested by cotransfection of HEK293T cells with epitope tagged expression vectors for Ubc3 and UbcH5 a, b and c. GST pulldown was used to extract and concentrate proteins associated with GST-RNF11, and an anti-GST Western blot shows that this protein was present in all the lysates. Ubc3 and three variants of UbcH5 were also found in lysates transfected with His-tagged protein expression vectors for each, as revealed by an anti-His tag Western blot of whole-cell lysates (Figure 4

). Interestingly, the His-tagged UbcH5 proteins, but not the His-Ubc3 protein, are seen on the Western blot made after GST pulldown of GST-RNF11. This indicates that RNF11 interacts with UbcH5s but not with Ubc3.

### Ubiquitination of RNF11 by Smurf2 requires the PY motif

**Figure 5:**
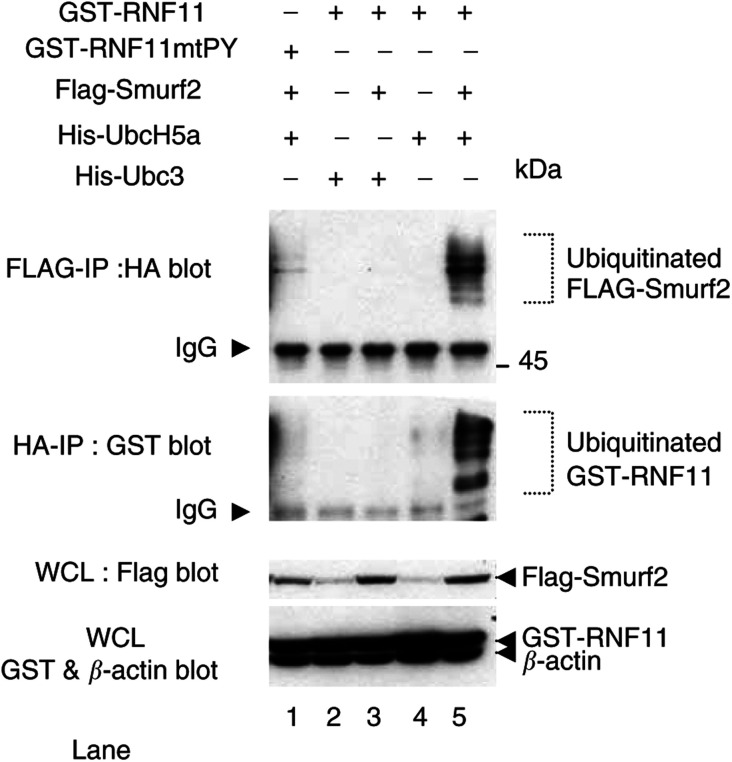
RNF11 is ubiquitinated in the presence of UbcH5a and Smurf2. HA, GST and FLAG Western blots of anti-HA and Flag immunoprecipitates or whole-cell lysates as indicated are shown. The cell lysates were prepared from HEK293T cells cotransfected with HA-tagged ubiquitin and various combinations of GST-RNF11 or its mutant GST-RNF11mtPY with Flag-tagged Smurf2, and/or His-tagged E2 constructs as indicated.

Smurf2 is a HECT-type E3 Ub ligase which suggests to us that RNF11 may be one of its targets for ubiquitination. This was tested by cotransfection of FLAG-Smurf2 and GST-RNF11 with HA-tagged Ub and Ubc3 or UbcH5 constructs followed by immunoprecipitation of ubiquitinated proteins with anti-HA or anti-Flag antibody and anti-GST or anti-HA Western blotting (Figure 5

). Anti-HA immunoblotting showed ubiquitination of Smurf2 and associated proteins immunoprecipitated by anti-Flag antibody when RNF11 and UbcH5a were cotransfected with Smurf2 (Figure 5). Weak bands resulting from cotransfection with the PY mutant of RNF11 suggest that this mutation severely reduces ubiquitination of Smurf2 (Figure 5, lane 1). An anti-GST Western blot of the same lysates also revealed bands corresponding to ubiquitinated GST-RNF11 in lysates of cells cotransfected with Flag-Smurf2 and UbcH5a (Figure 5, lane 5). In the absence of Ubc5a, Flag-Smurf2 protein was not sufficient to allow immunoprecipitation of either ubiquitinated RNF11 or ubiquitinated Smurf2 (Figure 5, lane 3). Similarly, no ubiquitinated proteins were seen on either blot in lysates transfected with Ubc3 (Figure 5, lanes 2 and 3). As expected, anti-FLAG and anti-GST reactive proteins were seen in whole-cell lysates transfected with constructs for FLAG-Smurf2 and GST-RNF11.

### RNF11 relieves Smurf2-mediated transcriptional inhibition of a TGF*β* responsive promoter

**Figure 6:**
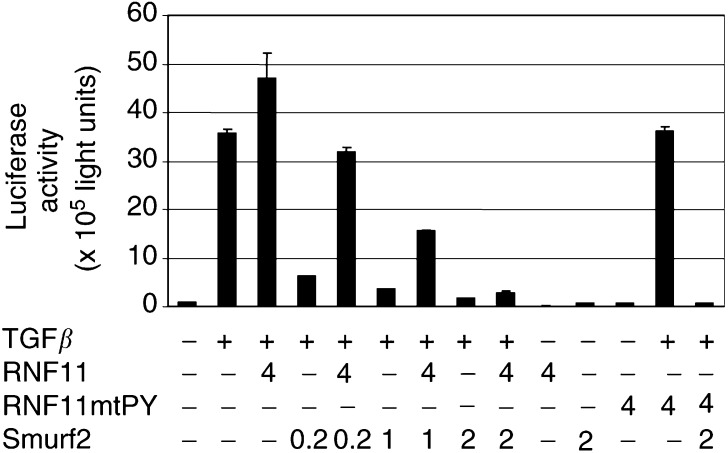
RNF11 affects TGF*β* responsive gene transcription. HepG2 cells were transiently transfected with 4 μg of p3TP-Lux alone, RNF11 or RNF11-*mt*PY mutant, and varying concentrations of Smurf2. Cells were incubated overnight in the absence or presence of 100 pM of TGF*β* and the relative luciferase activity was measured in cell lysates. Luciferase activity was normalised to *β*-galactosidase activity and is expressed as the mean±s.d. of triplicates from a representative experiment.

Cooperative interaction between Smurf2 and Smad7 results in inhibition of TGF*β* signalling by degradation of the TGF*β* receptor I (Kavsak *et al*, 2000). The inhibitory activity of Smurf2 is dependent on interaction of its WW domains with the PY motif of Smad7 (Kavsak *et al*, 2000). We have shown that the WW2 and WW3 domains of Smurf2 are necessary for interaction with RNF11 (Figure 3). RNF11 protein might therefore inhibit interactions between Smad7 and Smurf2, thereby affecting TGF*β* signal transduction. The effect of RNF11 on TGF*β*-dependent transcription activation was investigated by transient cotransfection of RNF11 and 3TP Lux promoter–reporter plasmids (Figure 6

). The 3TP Lux reporter vector contains a TGF*β* responsive portion of the plasminogen activator inhibitor (PAI) promoter linked to the coding region of the firefly luciferase gene. The PAI promoter is highly active in TGF*β*-treated cells and inactive in the absence of TGF*β* (Figure 6). We found that its reporter gene activity is reduced by more than seven-fold by cotransfection with the Smurf2 expression vector even in the presence of exogenous TGF*β* (Figure 6). Interestingly, reporter gene activity is restored when RNF11 is included in cotransfection with Smurf2. This relief of inhibition depends upon the wild-type PY motif in RNF11, as the RNF11 *mt*PY does not produce this effect when contransfected with the Smurf2 vector in TGF*β*-treated HepG2 human hepatoma cells.

## DISCUSSION

In this study, we chose the RNF11 gene for characterisation of its protein expression and mechanisms of action in normal and cancer cells because it was isolated from a tumour cell-enriched cDNA library, is unique, and contains modular domains and motifs known to interact with other proteins involved in oncogenesis. TMA technology allowed for a rapid and simultaneous analysis of RNF11 protein expression in 125 primary tumours microarrayed in duplicate by immunohistochemical staining using anti-RNF11 antibody (Kononen *et al*, 1998; Landberg *et al*, 2001). Comparison of RNF11 expression between breast, prostate, head and neck, kidney, colon and lung cancers revealed that the majority of our tumour array specimens produced clear immunostaining without any detectable background, and the morphology of the tissues was well preserved (Figure 1). To facilitate comparison of our results with published data, we employed the commonly used semiquantitative evaluation of protein expression based on distinct differences between strong, moderate and lack of staining in tumour specimens developed with immunoperoxidase methods (Kononen *et al*, 1998; Landberg *et al*, 2001). RNF11 was markedly overexpressed in breast and to a somewhat lesser extent in pancreatic as well as colon cancer samples, whereas it was only weakly expressed in prostate and renal cell carcinomas (Table 1, Figure 1).

The RNF11 gene was originally cloned as T2A10, a cDNA fragment from our library enriched for breast tumour-specific mRNAs. We found it to be identical to the human RNF11 protein, and it encodes modular domains and motifs known to interact with other proteins involved in oncogenesis (Kitching *et al*, 2003). Chief among these is the RING-H2-finger domain that could facilitate protein–protein interaction(s) leading to the degradation of specific substrate(s) involved in oncogenesis and the PY motif that could bind to WW domain proteins, including several HECT-type E3 ligases such as NEDD4, AIP4 and Smurf2. The PPPPY motif sequence of RNF11 is identical to that of Smads 2, 3 and 7, which have been shown to bind WW domains of Smurf2 Ub ligase and mediate the ubiquitination and degradation of various target proteins (Coopman *et al*, 2000; Longnecker *et al*, 2000). Smurf2 plays a key role in the ubiquitination and proteasomal degradation of receptor-activated Smad2 and Smad3, and corepressor SnoN (Bonni *et al*, 2001; Mizuide *et al*, 2003). The Smurf2 and Smad7 complex ubiquitinates T*β*RI, a key event resulting in degradation of the receptor and TGF*β* resistance in cancer cells (Kavsak *et al*, 2000).

Through site-directed mutagenesis, we showed that the tyrosine residue of the RNF11 PY motif is essential for binding to Smurf2 (Figure 2). Indeed, mutation of the PY motif, but not the RING-H2, eliminates the interaction with Smurf2 (Figure 2). We also investigated which of the Smurf2 WW domains might be necessary for interaction with the PY domain of RNF11 (Figure 3). Only wild-type Smurf2 and the mutant that contains the WW2 and WW3 domains were able to interact with RNF11, indicating that interaction of RNF11 with Smurf2 does not require the WW1 domain (Figure 3).

RNF11 binding to Smurf2 in mammalian cells suggests that, similar to Smads 2 and 3, it may also recruit targets for destruction by Smurf2 E3 ligase. RNF11 is a small RING-finger protein, similar to ROC1 and APC11, which are components of the SCF and the anaphase promoting complexes respectively (Chen *et al*, 2000; Jackson *et al*, 2000; Maeda *et al*, 2001). These two ubiquitin ligase complexes regulate Ub-mediated protein degradation during G1/S and anaphase. The importance of the small ring-finger proteins ROC1 and APC11 in these complexes suggests that RNF11 may also have a role in multi-subunit E3 complexes.

In general, ubiquitin moieties are transferred from an E2 to a catalysing ubiquitin protein ligase E3 and then on to the substrate protein. However, the E2 enzyme for the HECT-type ligase Smurf2 is unknown. Our results suggest that interaction of RNF11 with Smurf2 allows ubiquitination of Smurf2, and this is dependent upon the presence of UbcH5a (Figure 5). This suggests that RNF11 is required for the ubiquitination of Smurf2 that is essential to its ligase activity (Yagi *et al*, 1999). Interaction between UbcH5a and RNF11 was confirmed in a similar but separate experiment showing that UbcH5s, but not Ubc3, interacts with RNF11 in the absence of Smurf2 (Figure 4). RNF11 is therefore a PY motif containing RING-finger protein that may mediate activation of the HECT-type E3 ligase Smurf2 by the E2 conjugating UbcH5 enzymes (Figure 5).

Smurf2 also interacts with the PY motif of Smad7, forming a complex essential to the ubiquitination and degradation of T*β*RI. It has been reported that the Smad7 protein suppresses TGF*β* dependent induction of the 3TP-Lux reporter (Hayashi *et al*, 1997). Transactivation and association of Smurf2 with Smad7 further reduces this transcription activity due to degradation of the TGF*β* RI (Hayashi *et al*, 1997; Kavsak *et al*, 2000). We hypothesised that the PY motif of RNF11 could affect interactions with Smad7. This was tested by us in a transcription–transactivation assay similar to that used to illustrate Smad7/Smurf2 effects on TGF*β* responsive gene transcription (Hayashi *et al*, 1997; Kavsak *et al*, 2000). As expected, TGF*β* treatment induced 3TP-Lux reporter activity in transfected cells, and this transcriptional activity was inhibited by Smurf2 but not by RNF11 (Figure 6). However, the transcriptional activity of the reporter gene was restored when increasing amounts of RNF11 were used in conjunction with Smurf2 (Figure 6). This suggests that RNF11 may interfere with the binding of Smad7 to Smurf2, thus inhibiting the degradation of T*β*RI protein resulting in restoration of transcription of reporter gene activity. Moreover, the PY mutant of RNF11 which does not bind to Smurf2 is unable to restore the activation of the 3TP reporter. Thus, our data strongly support a role for RNF11 affecting the Smurf2/Smad7 protein–protein interactions involved in TGF*β* signalling. High-level expression of Smurf2 has been found in oesophageal squamous cell carcinoma (SCC) and seems to correlate with poor prognosis in these patients (Fukuchi *et al*, 2002). The expression pattern of RNF11 in human primary tumours is interesting when compared to Smurf2 in that head and neck tumours including oesophageal SCC samples expressed RNF11 in 63% of the surveyed cases. This is particularly relevant in the light of the observed overexpression of RNF11 in invasive breast cancers and its affects on TGF*β* responsive gene activation (Figures 1 and 6).

Although Smad proteins inhibit cell proliferation, they can also induce tumour invasion and metastasis. Similarly, in animal models it has been reported that TGF*β* can inhibit early stages of tumour development but stimulate later tumour progression and metastasis. The interaction of RNF11 and Smurf2 could be important here because it restores TGF*β* signalling and RNF11 could have multiple functions similar to Smad proteins. Perhaps, RNF11 blocks some of the inhibitory effects of Smad7 on TGF*β* signalling on cell proliferation and apoptosis, but leaves intact the positive effects of this pathway on malignant progression.

An abundance of regulatory proteins, including growth factors and their receptors, adaptor proteins involved in signal transduction, transcription factors and cell cycle regulating proteins, is coordinated by balancing their synthesis and degradation. As shown here, RNF11 interacts with ubiquitin E2 conjugating enzymes and E3 ligases and thus could potentially facilitate the degradation of specific substrate(s) involved in oncogenesis. The ongoing identification of target proteins that are ubiquitinated in the presence of RNF11 will allow us to determine the significance of its role in tumorigenesis (Li and Seth, 2003). In addition, we are investigating how its gene product can be used as a target for molecular diagnosis and therapeutic intervention of breast and other cancers.
